# Efficacy of Risk Prevention Management Based on Heinrich’s Law in Nursing Care of Elderly Patients Undergoing Digestive Endoscopy

**DOI:** 10.12669/pjms.40.11.10744

**Published:** 2024-12

**Authors:** Li Huang, Sujuan Zhu, Jing Chu, Suqin Zhong

**Affiliations:** 1 Li Huang Department of Endoscopy Center, Hai’an Traditional Chinese Medicine Hospital, Hai’an, Jiangsu Province 226000, P.R. China; 2 Sujuan Zhu Department of Gynecology, Hai’an Traditional Chinese Medicine Hospital, Hai’an, Jiangsu Province 226000, P.R. China; 3 Jing Chu Department of Orthopedics, Hai’an Traditional Chinese Medicine Hospital, Hai’an, Jiangsu Province 226000, P.R. China; 4 Suqin Zhong Department of Gastroenterology, Hai’an Traditional Chinese Medicine Hospital, Hai’an, Jiangsu Province 226000, P.R. China

**Keywords:** Heinrich’s Law, Risk prevention management, Elderly, Digestive endoscopy, Nursing Care

## Abstract

**Objective:**

To study the efficacy of risk prevention management based on Heinrich’s Law in nursing care of elderly patients undergoing digestive endoscopy (DE).

**Methods:**

In this retrospective study clinical data of 182 elderly patients who underwent DE in Hai’an Traditional Chinese Medicine Hospital from April 2021 to September 2023 were collected. A total of 89 patients received routine nursing care (Control-group), and 93 patients received risk prevention management based on Heinrich’s Law in addition to the routine nursing (Observation-group). Vital signs indicators, stress response levels, Boston bowel preparation score, State-Trait Anxiety Inventory-State (STAI-S), State-Trait Anxiety Inventory-Trait (STAI-T) score, incidence of adverse events, and nursing satisfaction were assessed and compared between the two groups.

**Results:**

Vital signs of the patients in the Observation-group were more stable than those of the Control-group 15 minutes and 30 minutes after the endoscopic inspection (*P*<0.05). After the procedure, stress response of the Observation-group was significantly milder than that of the Control-group (*P*<0.05). The Boston bowel preparation score in the Observation-group was significantly higher (*P*<0.05), while STAI-S and STAI-T scores were significantly lower than those in the Control-group (*P*<0.05). Implementing risk prevention management based on Heinrich’s Law was associated with significantly lower incidence of adverse events (*P*<0.05), and markedly higher nursing satisfaction (*P*<0.05).

**Conclusions:**

Adopting a risk prevention management model based on Heinrich’s Law for elderly patients undergoing DE can stabilize vital signs, alleviate anxiety, reduce the risk of adverse events, and increase patient satisfaction in this vulnerable group.

## INTRODUCTION

Digestive endoscopy (DE) is an important diagnostic technique for digestive system diseases.^1^ It is used to clarify the progression of small lesions in the duodenum, stomach, and esophagus through endoscopy.^2^ It not only detects the presence and the severity of the digestive tract lesions, but also allows to evaluate treatment outcomes.^1^-^3^ DE has advantages such as simplicity of the procedure and minimally invasive approach.^1^,^2^ However, it is still an invasive inspection methods. Elderly patients, in particular, may have difficulty understanding DE correctly and may be concerned about the inspection results. Therefore, this population of patients often present with serious anxiety, fear, and physiological stress reactions before the inspection.^4^,^5^ In addition, invasive nature of the procedure and medications that are used can cause discomfort, further exacerbating the degree of stress response, which not only affects the inspection results but may also lead to adverse events.^4^-^6^ Therefore, it is important to implement effective nursing interventions during DE in elderly patients.^7^

Routine nursing measures for DE mostly focus on explaining the inspection process and related precautions.^8^ However, lack of sufficient attention to the patient’s physical and mental state makes it difficult to achieve the overall clinical effect that would meet clinical expectations.^8^,^9^ Heinrich’s Law, proposed by Herbert W Heinrich, was first applied in the aviation industry for safety flight management. It emphasizes that every accident is the result of accumulation of various factors and can be prevented through regular safety inspections.^10^,^11^ Research has been conducted on effectiveness of nursing safety management in breast outpatient operation room based on Heinrich’s Law, and showed that such nursing approach can reduce the occurrence of adverse events in the operation room and improve the nursing quality and patient satisfaction.^10^,^12^ However, there are currently few reports and studies on the application value of Heinrich’s Law in DE in clinical practice. This study aimed to retrospectively analyze clinical data of elderly patients who underwent DE in our hospital to clarify the application value of risk prevention management based on Heinrich’s Law in nursing care of elderly patients undergoing DE.

## METHODS

Clinical data of 182 elderly patients who underwent DE in Hai’an Traditional Chinese Medicine Hospital from April 2021 to September 2023 were retrospectively selected. Among them, 89 patients received routine nursing care and were included as the Control-group, and 93 cases received risk prevention management based on Heinrich’s Law in addition to the routine nursing, and were assigned to the Observation-group.

### Ethical Approval:

The hospital’s ethics committee approved this study with the number HZYLL2023041, Date: October 05^th^ 2023.

### Inclusion criteria:

- Age ≥ 60 years old.
- Patients who met the indications for DE and underwent DE for the first time.
- Having good compliance and understanding, cognitive, and communication skills.
- Complete clinical data.

### Exclusion criteria:

- Patients with infectious diseases (such as tuberculosis, AIDS, Hepatitis-B, etc.).
- Patients with a tendency for systemic bleeding or gastrointestinal bleeding.
- Patients with inflammation in the esophagus, throat, oral cavity, etc.
- Patients with schizophrenia or consciousness disorders.
- Patients with severe respiratory and circulatory system diseases.

### Routine nursing care:

Routine care was carried out strictly following medical advice to implement DE related diagnosis, treatment, and nursing operations. Before the inspection, patients were instructed to strictly abstain from drinking water and eating food; history of allergies was recorded, and patients’ intestines were well prepared. During the inspection, all relevant operations were performed gently and slowly, and patients’ vital signs were monitored. Patients were instructed not to change their position arbitrarily; language and body movements were used to relieve patients’ tension and ensure that they could remain relaxed. Within two hours after the inspection, patients were instructed to fast and avoid coughing hard to prevent damage to the throat mucosa; After two hours, non-irritating food can be consumed.

### Risk prevention management based on Heinrich’s Law:

***Risk assessment:*** A detailed analysis of the DE process was done, and existing or potential risks were analyzed at each stage of the perioperative period. Ultimately, it was determined that main nursing risk events included patients not preparing their intestines as required, non-standard diagnostic and treatment environments, lack of aseptic awareness, and non-standard classification of related equipment.

***Improve management:*** Nursing staff brainstormed options to improve outcomes based on “DE safety management”. Proposed strategies were based on the types of nursing risk events, starting from the causes and protective measures, establishing a comprehensive and clear-thinking management, and covering all aspects of DE procedure. Nursing staff were strengthened with professional training, mainly including quality control of DE nursing operations, hospital infection prevention and control, disinfection and sterilization standards, nursing risk education, professional ethics education, basic skill training, and DE diagnosis and treatment knowledge training, to ensure that the nursing staff had a strong sense of responsibility and improved their skills and performance during the procedure. Nursing staff were also regularly tested and assessed to consolidate their professional skills and ensure standardization and accuracy in their operations.^13^

***Establish improvement measures:*** Patients were provided detailed information on the DE process and related precautions before the inspection. Patients were guided to make appointments at different time periods and to thoroughly study procedure-related general information, such as inspection types, and locations. DE process and precautions were explained through manuals, multimedia videos, or oral presentations, based on the patient’s educational background. Care was taken to use language that would be able to alleviate patients’ fear, tension, and anxiety. ii. Improving zoning management of endoscopic inspection rooms: various items and personnel were diverted through different channels. DE-related items, equipment, instruments, and drugs in each area were arranged according to regulations, to ensure reasonable layout, and to prevent cross infection. Care was taken to improve medical risk prevention and control management system, standardize specialized technical operations of nursing staff, and ensure that sterile items, disposable consumables, etc. can be traced and recorded from receipt to final disposal. iii. Strict adherence to sterile procedures during DE; training on standardized report writing for nursing staff, and face-to-face verification. Performance of the equipment was strictly evaluated before use to ensure that all equipment and instruments are in normal working condition. Patients’ psychological stress was relieved using physical touch, light music, and other forms to divert their attention. iv. After the inspection, patients were provided guidance on diet, emotional well-being, exercise, medication, and sleep. Patients were instructed on proper self-observation and emergency response measures.

### Observation indicators and evaluation criteria:

- Vital sign indicators were recorded before the procedure, 15 minutes and 30 minutes during the inspection, and after the inspection, and included respiratory rate, heart rate, and mean arterial pressure.
- Stress response levels before and after the inspection, including serum levels of adrenaline, norepinephrine, and cortisol. Levels of adrenaline and norepinephrine in serum were measured by high-performance liquid chromatography; cortisol levels were detected using radioimmunoassay. The reagent kit was purchased from Shanghai Biyotime Biotechnology Co., Ltd.
- Intestinal preparation, assessed by Boston Bowel ***Preparation Score***:^14^ The Boston scale divides colon into three segments: transverse colon, left colon, and right colon, with a total score of nine points and an average of three points per segment. Total score of ≥ six points and each segment score of ≥ two points indicated fully prepared colon.

### Anxiety before and after the inspection:

The State-Trait Anxiety Inventory (STAI) was used to evaluate the patient’s level of anxiety.^15^ STAI includes 40 items, with 20 items measuring State Anxiety (STAI-S) and 20 items measuring Trait Anxiety (STAI-T); STAI-S ≥ 55 indicates high state anxiety; A score of STAI - T ≥ 55 indicates high trait anxiety.

Adverse event incidence rate, including bed falls, falls, inadequate instrument preparation, and inadequate patient preparation.

### Nursing satisfaction:

Self-developed scale evaluation was used and included service attitude, professional ability, and nursing quality, with a total of 10 points. Based on the score, patient’s response was interpreted as very satisfied (9-10 points), satisfied (7-8 points), and dissatisfied (≤ 6 points). Very satisfied and satisfied were included in the total satisfaction.

### Statistical Analysis:

All data analyses were conducted using SPSS 20.0 software (IBM Corp, Armonk, NY, USA) and PRISM 8.0 software (GraphPad, San Diego, USA). Quantitative data were represented by mean ± standard deviation, independent sample *t*-test was used for inter group comparison, and paired *t*-test was used for intra group before and after comparison. Chi square test was used to represent the number of cases. *P*<0.05 indicated statistically significant difference.

## RESULTS

**Fig.1:**
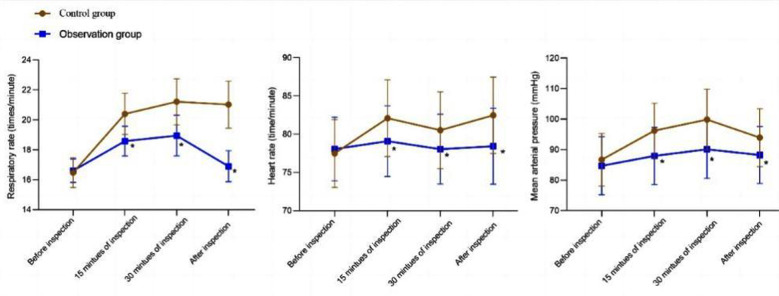
Changes in respiratory rate, heart rate, and mean arterial pressure of two groups of patients; ^*^P<0.05.

Clinical data of 182 patients met eligibility criteria of the study. Patients who received routine nursing care comprised the Control-group (n=89), and patients who received routine care combined with risk prevention management nursing based on Heinrich’s Law (n=93) were assigned into the Observation-group. There was no significant difference in general information between the two groups of patients (*P*>0.05). Table-I The respiratory rate, heart rate, and mean arterial pressure of patients in the Observation-group were significantly lower than those in the Control-group at 15 minutes, 30 minutes, and after the inspection (*P*<0.05). Fig.1

**Table-I:** Comparison of baseline characteristics between the two groups.

Item	Control group (n=89)	Observation-group (n=93)	χ^2^/t	P
Gender (male/female)	61/28	52/41	3.080	0.079
Age (year)	68.79±4.49	67.76±4.47	1.541	0.125
BMI (kg/m²)	22.75±2.82	23.19±3.36	-0.966	0.335
Disease duration (days)	3.13±1.56	3.45±1.66	-1.326	0.187
Education level			1.339	0.247
Below High School	60 (67.42)	55 (59.14)		
High school and above	29 (32.58)	38 (40.86)		
Disease type			0.976	0.614
Abdominal pain	25 (28.09)	21 (22.58)		
Rectal bleeding	24 (26.97)	30 (32.26)		
Changes in stool characteristics	40 (44.94)	42 (45.16)		

**Fig.2:**
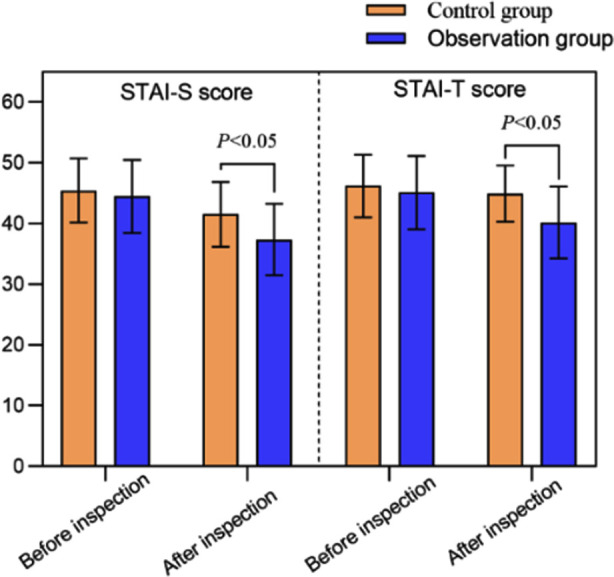
Comparison of STAI-S and STAI-T scores between two groups.

After the procedure, levels of adrenaline, norepinephrine, and cortisol in the Observation-group were significantly lower than those in the Control-group (*P*<0.05). Table-II The Boston bowel preparation scores of the Observation-group patients were significantly higher compared to the Control-group (*P*<0.05). Table-III Post-DE STAI-S and STAI-T scores of the Observation-group were significantly lower than those of the Control-group (*P*<0.05). Fig.2 The incidence of adverse events in the Observation-group (3.23%) was lower than that in the Control-group (11.24%) (P<0.05). Table-IV. The nursing satisfaction of the Observation-group (94.62%) was higher than that of the Control-group (80.90%) (P<0.05). Table-V

**Table-II:** Comparison of stress response levels between two groups.

Group	Adrenaline (nmol/L)		Norepinephrine (nmol/L)		Cortisol (ng/L)	

	Before inspection	After inspection	Before inspection	After inspection	Before inspection	After inspection
Control-group (n=89)	0.58±0.12	0.84±0.14	1.17±0.18	1.39±0.18	127.61±18.46	140.70±19.13
Observation-group (n=93)	0.59±0.14	0.65±0.13	1.22±0.19	1.24±0.20	122.96±19.66	129.72±2054
*t*	-0.345	9.448	-1.588	5.257	1.644	3.727
*P*	0.731	<0.001	0.114	<0.001	0.102	<0.001

**Table-III:** Comparison of Boston Bowel Preparation Scores between Two Group.

Group	Right colon score	Transverse colon score	Left colon score	Total score
Control-group (n=89)	1.93±0.58	2.09±0.54	2.01±0.51	6.03±0.80
Observation-group (n=93)	2.33±0.60	2.54±0.50	2.60±0.55	7.47±1.09
*t*	-4.598	-5.814	-7.480	-10.172
*P*	<0.001	<0.001	<0.001	<0.001

**Table-IV:** Comparison of incidence rates of adverse events between two groups.

Group	Falling down from bed	Fall	Insufficient instrument preparation	Insufficient patient preparation	Total incidence rate (%)
Control-group (n=89)	1 (1.12)	3 (3.37)	2 (2.25)	4 (4.49)	10 (11.24)
Observation-group (n=93)	0 (0.00)	0 (0.00)	1 (1.08)	2 (2.15)	3 (3.23)
*χ^2^*					4.399
*P*					0.036

**Table-V:** Comparison of nursing satisfaction between two groups.

Group	Very satisfied	Satisfied	Dissatisfied	Total Satisfaction (%)
Control-group (n=89)	41 (46.07)	31 (34.83)	17 (19.10)	72 (80.90)
Observation-group (n=93)	54 (58.06)	34 (36.56)	5 (5.38)	88 (94.62)
*χ^2^*				8.061
*P*				0.005

## DISCUSSION

This study confirmed that the application of a risk prevention management plan based on the Heinrich’s Law in elderly patients undergoing DE can achieve good results, and is more efficient than routine care alone in stabilizing patient’s vital sign indicators, reducing their psychological stress level. Risk prevention management based on Heinrich’s Law in addition to the routine nursing effectively encouraged patient to maintain a good physical and mental state, reduced DE-associated risk of adverse events, and ensured overall effectiveness and safety of DE.

The Heinrich’s Law, an important safety production and management law, states that all adverse events result from the accumulation of hidden danger factors. Therefore, timely early identification and elimination of these potential risk factors ultimately lowers the incidence of adverse events.^11^,^12^ At present, the clinical value of Heinrich’s Law has been partially confirmed by research. Xue Guiping^16^ implemented risk prevention management based on the Heinrich’s Law, and showed that this intervention measure effectively reduces the incidence of nursing management problems and disputes, and improve the overall quality of nursing management in the emergency department. Wang Y et al.^17^ used the Heinrich’s Law in the safety management of operating room nursing, and demonstrated that it can improve teaching ability, comprehensive rescue ability, specialized theoretical knowledge, and single emergency operation ability of operating room nursing staff. After implementing the Heinrich’s Law, the nursing personnel started identifying their work shortcomings more actively, noticed improvement in language and behavior, promptly and accurately executed medical orders, and had reported significantly improved skills in operation and active cooperation.

Similar to the study by Wang Fang,^13^ our study also confirmed the application value of Heinrich’s Law in elderly patients undergoing DE. Our study further confirmed that the Heinrich’s Law is beneficial for simplifying the process of DE and is associated with reduced occurrence of adverse events.^12^,^13^,^16^,^17^ The workload of the endoscopic inspection room is substantial, with high variability in patient populations. Nursing staff often lack awareness of aseptic techniques and the diagnosis and treatment environment is not standardized.^18^ Since the Heinrich’s Law focuses on implementing corresponding interventions based on these factors, it ultimately enhances risk prevention and control awareness of nursing staff, and allows to avoid safety hazards. 12,13,16,17 Risk prevention management focuses on improving the quality of specialized nursing services, conducting targeted assessments of nursing risk factors, and promoting nursing staff to shift from passive to active care.^19^ During the perioperative inspection period, comprehensive education on cooperation skills and precautions should be implemented to alleviate patient’s anxiety and resistance to lower the degree of psychological stress response.^20^

Warning mechanism based on the Heinrich’s Law can shorten the time required for inspection and gastrointestinal function recovery, reduce psychological stress, and improve physical and mental comfort.^21^ We may speculate that nursing staff can refer to the Heinrich’s Law to implement risk warning assessment on relevant work processes, analyze existing and potential problems at every stage of the procedure. That allows to implement appropriate early warning interventions with a particular focus on nurse patient communication during the preoperative evaluation and preparation stages, such as implementing phased appointments and providing psychological support. Such approach will not only improve the inspection time, but also contribute to alleviating patient’s negative emotions and improve their psychological endurance.^21^,^22^

The results of this study also showed that the nursing satisfaction of the Observation-group was higher than that of the Control-group (*P*<0.05). It is plausible that risk prevention management model based on Heinrich’s Law improves the acknowledgement and appreciation of nursing work among elderly patients undergoing DE. Risk prevention management model based on the Heinrich’s Law encourages nursing staff to assess the deficiencies in the perioperative inspection period of DE and develop targeted improvement measures. That would ultimately lead to shorter waiting times, and alleviate procedure-associated their fear and anxiety of elderly patients.^23^,^24^

### Limitation:

This is a single-center retrospective analysis, with a small sample size and selection bias. Additionally, while this study focused on analyzing the risk prevention management based on the Heinrich’s Law during DE, elderly patients undergoing DE were not evaluated by endoscopists. Subsequent large-scale studies are needed to validate the results of this study.

## CONCLUSION

In elderly patients undergoing DE, risk prevention management based on the Heinrich’s Law in nursing care can more efficiently stabilize vital signs, reduce stress response, improve intestinal preparation, alleviate anxiety, reduce the risk of adverse events, and increase overall satisfaction compared to routine nursing approach.

### Authors’ contributions:

**LH:** Study design. literature search, manuscript writing.

**SZ**, **JC** and **Suqin Zhong:** Contributed to the data collection, analysis, interpretation and critical review.

**Suqin Zhong:** Literature search, data analysis, Critical review

All authors have read, approved the final manuscript and are responsible for the integrity of the study.
